# Optimization of experimental conditions for skin-PAMPA measurements

**DOI:** 10.5599/admet.761

**Published:** 2020-03-04

**Authors:** Sara Soriano-Meseguer, Elisabet Fuguet, Adriana Port, Martí Rosés

**Affiliations:** 1 Departament de Química Analítica i Institut de Biomedicina, Universitat de Barcelona, Martí i Franquès 1-11, 08028 Barcelona, Spain; 2 Serra Húnter Programme, Generalitat de Catalunya, 08002 Barcelona, Spain; 3 ESTEVE Pharmaceuticals, Drug Discovery and Preclinical Development, Parc Científic de Barcelona, Baldiri Reixac 4-8, 08028 Barcelona, Spain

**Keywords:** Skin-PAMPA, Permeation, Optimization, Membrane retention, Membrane stability

## Abstract

In recent years, the parallel artificial membrane permeability assay (PAMPA) has been extended for prediction of skin permeation by developing an artificial membrane which mimics the stratum corneum structure, skin-PAMPA. In the present work, the different parameters affecting skin-PAMPA permeability, such as incubation time and stirring, have been studied to establish ideal assay conditions to generate quality data for a screening of active pharmaceutical ingredients (API) in early stage drug discovery. Another important parameter is membrane retention, which shows dependence on lipophilicity when compounds are in their neutral form. Furthermore, the stability of the membrane has been investigated at different pH values, especially at basic pHs. Finally, a good correlation between human skin permeability and skin-PAMPA permeability, with a large dataset (n = 46), has been established. The optimized assay conditions were an incubation time of 4 hours with stirring in a pH below 8. With all these considerations the thickness of the aqueous boundary layer is decreased as much as possible and the membrane stability is guaranteed.

## Introduction

Transdermal administration is considered as an alternative route to conventional oral delivery of drugs. Transdermal delivery offers significant advantages over oral administration: minimal first-pass metabolism, avoidance of the adverse effects in the gastrointestinal environment and the ability to provide a controlled and prolonged drug release [1]. Despite these advantages, the structure of the skin mainly composed of the stratum corneum (SC), the outermost layer which acts as the main penetration barrier, the viable epidermis and dermis, imposes a clear obstacle to the topical delivery of drugs into the systemic bloodstream.

Estimation of skin permeation has become crucial in the pharmaceutical and cosmetic industries. Among the *in vitro* models used to determine the skin permeation, Franz diffusion cell method applying human skin as a membrane is the most relevant [2], but this method is laborious, costly and ethically questionable. Besides, it suffers low intra- and inter-laboratory reproducibility. The parallel artificial membrane permeability assay (PAMPA) was developed by Kansy and co-workers [3] for the fast determination of the permeability through passive diffusion. This technique is crucial in the early stage of drug discovery and it has many advantages like low cost and high-throughput. The first published models allowed the prediction of gastrointestinal absorption (GIT-PAMPA) [4–6]. Then, other models were published for modelling the blood-brain barrier [7] and also for the estimation of skin permeation [8]. Later, Sinkó and co-workers developed the skin-PAMPA methodology consisting of a new membrane containing synthetic certramides, analogues of the ceramides found in the stratum corneum [9, 10]. Certramides are cheaper alternatives to natural ceramides with the potential to prolong the storage time. Although certramides are structurally different from ceramides, their comparable molecular mass and hydrogen bond acceptor/donor capacity enables them to act as the lipid constituents in the PAMPA sandwich membrane, together with cholesterol, stearic acid and silicone oil [9, 10]. Skin-PAMPA is a model that offers high reproducibility, is more cost-effective and less laborious than other *in vitro* skin experiments, and has demonstrated a high prediction capability with a good correlation with the human skin penetration data [10].

GIT PAMPA assay conditions such as permeation time, assay pH, stirring, use of cosolvents and selection of detection techniques have been studied during years and optimized to generate high quality and relevant data [5]. For skin-PAMPA, only a few articles have been published [10, 11] and most of them deal with liquid or semi-solid formulations [12, 13] and transdermal patches [14]. However, permeability studies of compounds in solution are very important when the permeation properties of API are investigated. The protocol of these studies depends on the physico-chemical parameters of the API and the composition of the membrane. Since the skin-PAMPA membrane contains different components and is more resistive compared to other PAMPA membranes, protocol differences as incubation time are expected. The reason for this is that PAMPA membranes are composed mostly of lipophilic components and hence lipophilic compounds can cross them quickly. However, skin-PAMPA consists of lipophilic and hydrophilic moieties, so permeable compounds are likely to cross both lipophilic and hydrophilic domains [10]. Another parameter that also depends on the composition membrane and physico-chemical properties of the API is the unstirred water layer (UWL), which is formed around both sides of the lipophilic membrane. The permeation process in PAMPA assays may be limited by the UWL, especially for lipophilic compounds. The water layer can act as rate-limiting transport giving smaller permeability values. To solve this, the stirring decreases the UWL thickness and thus the resistance of the water layer less of a contribution to the measured permeability [15]. The use of the oblate stir disks (flippers), rotating in a horizontal axis parallel to the plane of the microtitre plates, proved to be the most efficient stirring mechanism ever reported in microtitre plate permeation assays [15].

Another feature to consider is the stability of the membrane. Human skin pH is normally slightly acidic, values between 4 and 6, while the body’s internal environment mainly maintains a neutral pH. Fatty acids found in the lipid bilayer exist in the neutral or ionic forms depending on the pH, so they also contribute to the ionization of the membrane. At the SC surface, pH 5 causes minimal head‐group repulsion and promotes a bilayer structure. The pH 7 in the innermost SC layers produces 90% ionization of the fatty acids leading to head‐group repulsion. An increase of the pH leads to increase head‐group repulsion, disturbing epidermal lipid lamella and thus impairing barrier function [16]. As most of the drugs are weak acids or weak bases, it is relevant to study the influence of the pH on permeability. To this end, it is necessary to study the stability of the skin-PAMPA membrane at different pH values, especially at basic pHs.

In order to establish the optimal assay conditions to generate quality data for the screening of API in early stage drug discovery, the purposes of this work are to study the different parameters affecting skin-PAMPA permeability, such as incubation time and stirring, and also to check the stability of the membrane with pH.

## Materials and methods

### Reagents

Acetonitrile LiChrosolv grade was purchased from Merck (Darmstadt, Germany). Formic acid was obtained from Scharlau (Sentmenat, Spain). Dimethylsulphoxide was from Carlo Erba (Milano, Italy). Water was purified by a Milli-Q deionizing system from Millipore (Billerica, MA, USA) with a resistivity of 18.2 MΩ. Most solutes employed were purchased from Sigma-Aldrich (Steinheim, Germany), Fluka Analytical VWR (West Chester, PA, USA), Riedel-de Haën (Seelze, Germany), Merck (Darmstadt, Germany), Carlo Erba (Milano, Italy) and Baker (Center Valley, PA, USA). Some drugs were synthesized in ESTEVE (Barcelona, Spain).

The concentrated PRISMA HT^TM^ solution was used to prepare the buffer solutions. This solution is a universal buffer designed by Pion Inc (Billerica, MA, USA) and is formed by several compounds with p*K*_a_ values evenly spaced to produce a constant buffer capacity in the range pH 3-10. The ionic strength of the PRISMA HT^TM^ is about 10 mM. A hydration solution from Pion Inc. (Billerica, MA, USA) was used to rehydrate the artificial skin membrane.

The skin-PAMPA plates, with a membrane composed by certramides, cholesterol, stearic acid, and silicone oil, were also obtained from Pion Inc. (Billerica, MA, USA).

### Instruments

pH measurements were done with a combined Crison 5202 electrode in a Crison 2001 pH meter (Hach Lange Spain, L’Hospitalet de Llobregat, Spain). The electrode system was calibrated with the ordinary aqueous buffers of pH 4.01 and 7.00 (25 °C).

Permeability measurements were made with the PAMPA Explorer Permeability Assay instrument from Pion Inc (Billerica, MA, USA). This instrument is composed of the Gut-Box^TM^ and the TempPlate. The Gut-Box^TM^ is a mechanical device used for the PAMPA assay to decrease the permeation time and reduce the unstirred water layer (UWL) thickness that is always present. The TempPlate is used for the temperature control during plate incubation.

Chromatographic measurements were performed with a Waters (Milford, MA, USA) I-Class UPLC with diode array detector. Instrument control and processing was performed by Empower. The column used for the determinations was an Acquity UPLC BEH C18 (50 x 2.1 mm, 1.7 μm).

### Skin-PAMPA method

Before permeation assays, the top part of the skin-PAMPA sandwich, which contains the membrane, was hydrated overnight with the hydration solution. The samples were dissolved in diluted PRISMA HT^TM^ buffer solution at several pH values: 25 mL of concentrated PRISMA HT^TM^ was diluted with water to a final volume of 1 L and then, different solutions were prepared by pH adjustment between 3 and 10 with 0.5 M NaOH (Merck). The concentration of the sample solutions was 50 μM (containing 0.5% v/v DMSO). Skin-PAMPA assays were carried out under gradient-pH conditions to mimic the pH change between the stratum corneum and the underlying epidermis and dermis. For this reason, the donor compartment pH was varied from 3 to 10 and the acceptor compartment pH was maintained at pH 7.4. This gradient-pH state is the first sink condition in skin-PAMPA. The double-sink condition that is usually used in GIT-PAMPA consists in taking advantage of chemical scavengers in the receiver compartment to make permeation of lipophilic compounds across the membrane unidirectional. This procedure simulates the situation present in the body where blood flow and serum proteins constantly shift the concentration gradient to favor absorption. However, this double-sink condition is not used in skin-PAMPA since this additional shift is not observed through the skin. Before performing the skin-PAMPA sandwich, the donor compartment (or bottom plate) was prefilled with 180 μL (stirred assay) or 200 μL (unstirred assay) of sample solutions and the acceptor compartment (or top plate) was filled with 200 μL of PRISMA HT^TM^ buffer solution at pH 7.4. The donor volume is decreased in stirred assay since the stirring bars have a volume of 20 μL that can cause overflow. As it is mentioned before, the Gut-Box^TM^ was used to stir effectively.

The skin-PAMPA sandwich was incubated at 32 °C. After the permeation time was reached, the plates were separated and the compound concentration in acceptor, donor and reference (initial sample solution) was determined using UPLC-DAD. Chromatographic conditions were: formic acid 0.1% and acetonitrile as mobile phase, flow rate of 0.8 mL/min, linear gradient elution (linear gradient from 2% to 98% of acetonitrile in 2.5 minutes), injection volume of 5 μL and the detection by DAD. 3 to 5 replicate measurements were done per compound and pH, and every well-plate contained only one compound.

### Calculation methods

The skin-PAMPA permeability was calculated through PAMPA equations. Taking into account the membrane retention (mole fraction of the sample that can be lost in the membrane) under gradient-pH conditions, the equations are the following [17]:


(1)

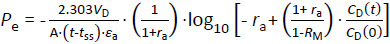




(2)

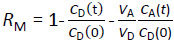



where *P*_e_ is the effective permeability coefficient (cm/s), *V*_D_ and *V*_A_ are the volume of solution in the donor side (180 μL stirred assay or 200 μL unstirred assay) and acceptor side (200 μL), respectively, *A* is the membrane area (0.3 cm^2^), *t* is the incubation time of the experiment (s), *τ*_ss_ is the lag time (s) [*τ*_ss_=(54·*R*_M_+1)·60 s], *ε*_a_ is the apparent membrane porosity (0.76), *C*_D_(*t*) is the concentration in the donor side at time *t*, *C*_D_(0) is the initial concentration in the donor side, *C*_A_(*t*) is the concentration in the acceptor side at time *t*, *R*_M_ is the membrane retention and *r*_a_ is the sink asymmetry ratio (gradient-pH-induced), defined as:


(3)

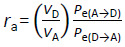



When the pH is different in the two sides of the membrane, a gradient-pH is created and the permeation of ionizable molecules can be altered. This gradient-pH implies two different permeability coefficients, one denoted by the superscript (D→A), associated with donor to acceptor flux, and the other denoted by the superscript (A→D), corresponding to the reverse-direction flux. As equation (3) has two unknowns, *P*_e(A→D)_ and *P*_e(D→A)_, the following method is used to solve the equation: at least two assays are done, one with gradient-pH and the other with iso-pH, that is, the same pH at both compartments. For iso-pH, *P*_e(A→D)_ = *P*_e(D→A)_. Therefore, *P*_e(A→D)_ can be solved directly using the iso-pH equation:


(4)

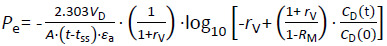



where *r*_v_ is the aqueous compartment volume ratio, defined as:


(5)

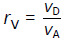



Then, Eq. (1) is iteratively solved for *P*_e(D→A)_. Initially, *r*_a_ is assumed to be *r*_v_, but with each iteration, the *r*_a_ estimation is improved by using the calculated *P*_e(D→A)_. The process continues until self-consistency is reached within the precision required (0.001). The Solver utility from Microsoft Excel was used for the iterative process.

## Results and discussion

### Optimization of skin-PAMPA assay conditions: incubation time and stirring

To evaluate the different parameters that affect skin-PAMPA permeability and to establish general assay conditions for skin-PAMPA determinations in solution, the permeability of 9 drugs with different acid-base properties, lipophilicity, and *in vitro* Franz Cell human skin permeability values (*K*_p_, units in cm/s) was investigated. To assure the neutral form, acidic compounds (flurbiprofen, ibuprofen, naproxen and 5-fluorouracil) were dissolved in PRISMA HT^TM^ buffer solution at pH 3.0. Aminopyrine, a basic compound with a aqueous p*K*_a_ value of 5, was dissolved in the buffer solution at pH 7.0. The neutral compounds (progesterone, griseofulvin, digitoxin and hydrocortisone) were dissolved at pH 3.0. The skin-PAMPA sandwiches were incubated (with and without stirring) for 30 min, 4 h and 24 h. The logarithm of skin-PAMPA permeability values (log *P*_e_) and the corresponding membrane retention are presented in Table 1. This table also shows the logarithm of the octanol-water partition coefficient (log *P*_o/w_) and the logarithm of human skin permeation coefficient (log *K*_p_) for each compound. The log *K*_p_ data was obtained from Zhang *et al.* database [18]. This database provides an extensive and carefully examined data set, where experimental log *K*_p_ data from literature is corrected for ionization in water and for the temperature at 37 °C.

**Figure 1.**
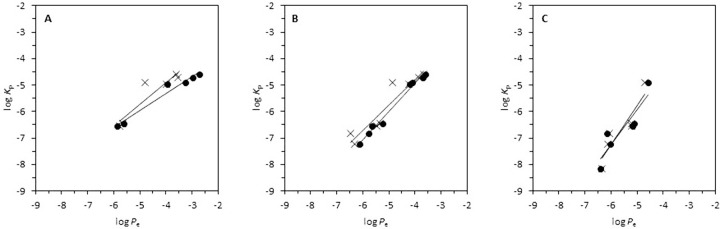
Effect of incubation time and stirring in the determination of the skin permeability (log *K*_p_) through the skin-PAMPA permeability (log *P*_e_). Incubation time: 30 minutes (**A**); 4 hours (**B**); 24 hours (**C**); stirred assay (●); non-stirred assay (⨉).

First, to study the effect of the incubation time and stirring in the determination of the skin permeability through the skin-PAMPA permeability, the log *K*_p_ values were correlated to the log *P*_e_ obtained from skin-PAMPA assays at each incubation time. The correlations obtained are presented in Figure 1 A-C. Each figure contains two different correlations, one for the stirred assays and another for unstirred assays. As it can be observed, the number of compounds that can be determined in 4 hours (n=8) is greater than in 30 minutes (n=6) and 24 hours (n=6). For some compounds, such as 5-fluorouracil, hydrocortisone and digitoxin, whose log *K*_p_ values are quite low, an incubation time of 30 minutes is not enough to reach the steady state, therefore their skin-PAMPA permeability values cannot be evaluated. After 4 hours of incubation, all drugs can be determined except digitoxin which does not arrive at the steady state. Digitoxin is considered very little permeable due to its very low *in vitro* skin permeability value (log *K*_p_ = -8.15). After 24 hours of incubation, digitoxin can reach the steady state and its skin-PAMPA permeability values can be determined. However, compounds such as flurbiprofen, ibuprofen and naproxen (with high log *K*_p_ values) cannot be determined. When a compound is highly permeable, long incubation times under gradient-pH conditions provoke that the donor and acceptor compartment concentrations achieve equilibrium values and hence the whole sample of the donor compartment moves to acceptor compartment due to sink conditions, making difficult to determine the permeability values.

Relative to stirring and non-stirring experiments, Table 1 shows the results obtained at different incubation times. At 30 minutes of incubation time flurbiprofen, progesterone and ibuprofen show great log *P*_e_ values in stirred assays compared to unstirred ones. This difference can be attributed to the presence of UWL due to the lipophilic character of the compounds (log *P*_o/w_ > 3). In this case, UWL acts as rate-limiting transport giving smaller permeability values. At 4 hours of incubation, log *P*_e_ values obtained from experiments with and without stirring are almost the same except for progesterone (log *P*_e_ equal to – 4.11 and -4.88, respectively) and 5-fluorouracil (log *P*_e_ equal to - 5.77 and -6.47, respectively). For progesterone, this difference can be justified by the presence of UWL or the high membrane retention values. The reason for 5-fluorouracil is unknown since it is a very hydrophilic compound and therefore the difference of values cannot be attributed to UWL effect. In general, it seems that with 4 hours of incubation time the aqueous boundary layer does not have much effect in most compounds. At 24 hours, apart from flurbiprofen, ibuprofen, and naproxen which couldn’t be evaluated, the results obtained with and without stirring are practically the same.

The membrane retention values can sometimes be very high depending on the composition of the PAMPA membrane. For example, membranes made of 2% DOPC (dioleoylphosphatidylcholine) dissolved in dodecane can have *R*_M_ values higher than 0.80 (*4*). In the case of the skin-PAMPA membrane, retention is in general low and goes from 0 to 0.30 for most of the analyzed compounds (see Table 2), except for progesterone whose values are very high (0.33-0.88). For some compounds this parameter can depend on the incubation time. For 5-fluorouracil, aminopyrine, and hydrocortisone this parameter is almost null; minimum retention is observed only after 24 h of incubation. Note that these compounds are quite hydrophilic and are almost neutral at the pH of the determination. Instead, for some other compounds such as digitoxin, griseofulvin and progesterone (all neutral compounds but with higher log *P*_o/w_ values) R_M_ clearly increases with incubation time. In the case of flurbiprofen, ibuprofen and naproxen, *R*_M_ decreases as the incubation time increases. These compounds are completely ionized at the pH of the acceptor compartment, and after some time they are all accumulated in there, showing negligible retention in the membrane. In general terms, this factor is independent of stirring or not the solutions as observed for all compounds except for progesterone, which retention factor increases with the stirring use.

The membrane retention values at 24 h incubation time have been correlated to the log *P*_o/w_ to check if membrane retention is related to lipophilicity. Flurbiprofen, ibuprofen, and naproxen are excluded from the correlation due to the reasons above mentioned. A sigmoidal relationship between *R*_M_ and log *P*_o/w_, which can be explained by equation 6 (see appendix), can be observed for the rest of the drugs, which are mainly in its neutral form.


(6)

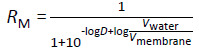



**Figure 2.**
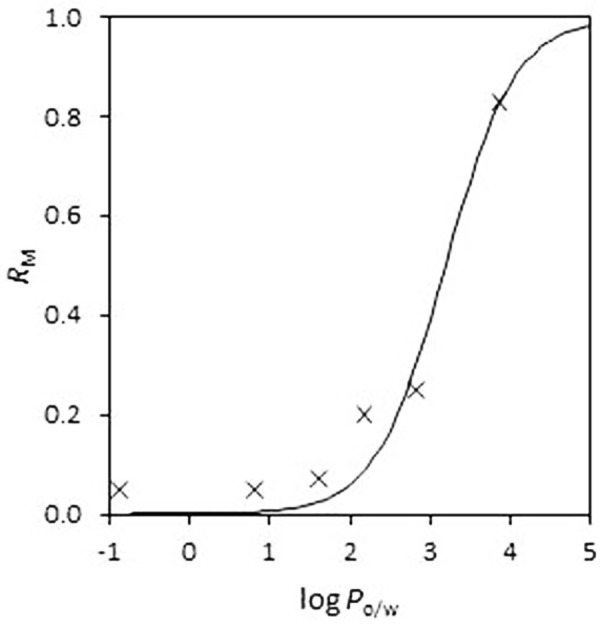
Relationship between membrane retention (RM) and lipophilicity (log Po/w) at 24 h incubation time without stirring.

In this equation *D* is the distribution coefficient between water and membrane (pH dependent), *V*_water_ is the volume of the aqueous compartment, and *V*_membrane_ is the volume of the membrane. Figure 2 shows the fit of equation 6 (using *P*_o/w_ of the compounds instead of log *D*) to the experimental data (24 h incubation time without stirring). Good statistics are obtained (R^2^ = 0.951, SD = 0.066, F = 98), with a value of 3.19 ± 0.11 for the parameter log (*V*_water_/*V*_membrane_). This is a good fit given that the true equilibrium may not have been achieved, ionisation of some compounds has been neglected, and octanol is expected to be more lipophilic than the membrane.

From correlations established in Figure 1 A-C, it can be observed that the slopes are very close and very low data dispersion is observed. Hence, the assay conditions using an incubation time of 4 hours (Figure 1B) and taking advantage of stirring to decrease as much as possible the thickness of the aqueous boundary layer are considered the most appropriate. Working under these conditions permits to evaluate the greatest number of compounds, despite in some cases, such as digitoxin, longer incubation might be needed.

### Study of membrane stability

**Figure 3.**
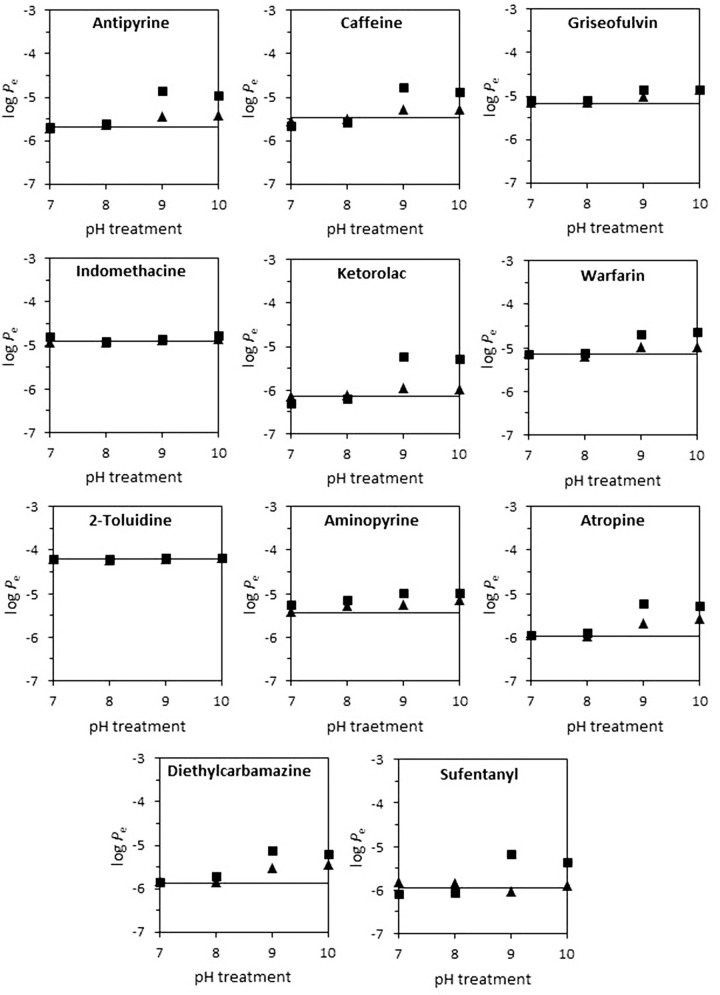
Influence of treatment time and pH in skin-PAMPA permeability. Treatment time: 30 minutes (▲); 4 hours (■).

The stability of the skin-PAMPA membrane at basic pH values was studied through measurement of the permeability of 11 compounds (3 acids, 5 bases and 3 neutrals). The skin-PAMPA membrane was hydrated with diluted PRISMA HT^TM^ buffer solution at pH 7, 8, 9 and 10 during 30 min and 4 hours, to be consistent with incubation times selected in section 3.1. Then, a standard permeation assay was carried out with an incubation time of 4 h and stirring at pH 5 in the donor compartment and pH 7.4 in the acceptor compartment. Simultaneously, permeation assays were also performed without the previous soaking treatment at basic pH. The obtained results are presented in Figure 3, which shows the skin-PAMPA log *P*_e_ values obtained with and without the previous treatment. Each subfigure corresponds to a compound and the straight line inside the figure is the log *P*_e_ value in the untreated membrane.

An increase of permeability values at pH 9 and 10 is shown in Figure 3 for almost all the compounds, which points out the lack of stability of the membrane in this pH range. In some cases, such as aminopyrine, some distortion is already noted at pH 8. As described in the literature [16], solutions at pH 7 or higher produce a change of membrane packaging in human skin. However, in the skin-PAMPA membrane loose of stability is not observed up to pH 8, probably due to the different membrane composition. In most cases, the permeability values increase for both 30 minutes and 4 hours of treatment, meaning that at 30 minutes the stability of the membrane is already altered by the basic pH. Griseofulvin and warfarin have a slight increase at pH 9 and 10. The values of indomethacin, 2-toluidine and sufentanyl, however, remain constant. As a general trend, it seems that this problem is more important when the compound is less permeable (log *P*_e_ below -5) regardless of the nature and ionization of the compound. In conclusion, although the PRISMA HT^TM^ universal buffer solution suggests a working pH range between 3 and 10, it is advisable not to exceed pH 8 to avoid damaging the skin-PAMPA membrane. In that cases where permeability must be determined at pH values higher than eight (for example to determine the log *P*_e_ of the neutral forms of bases with p*K*_a_ values higher than 7) alternative methods should be used, such the estimation equations proposed by Zhang *et al*. [18] or estimation through chromatographic measurements [24].

### Correlation with human skin permeation data

**Figure 4.**
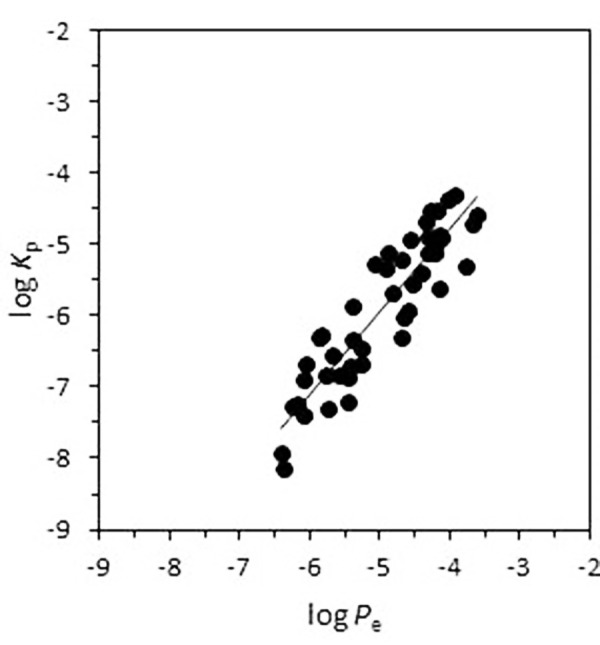
Correlation between human skin permeability and skin-PAMPA permeability.

The skin-PAMPA permeability (*P*_e_) of a large acid-base drugs dataset (n=46) obtained in the optimized assay conditions previously discussed has been correlated with literature skin permeability (*K*_p_). The log *K*_p_ data were collected from Zhang *et. al*. database [18]. Table 3 shows the log *K*_p_ and log *P*_e_ values of the compounds used in the correlation. Each compound was measured at the pH corresponding to the neutral form. Figure 4 plots the log *K*_p_ vs. the log *P*_e_ values whose correlation is presented in Eq. (7):


(7)

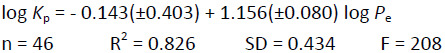



A good correlation between human skin and skin-PAMPA data and adequate statistical parameters have been obtained.

## Conclusions

The tests performed in this work indicate that the ideal assay conditions for skin-PAMPA permeability measurements are 4 hours of incubation time and with the use of stirring. This incubation time allows the determination of permeability of the greatest number of compounds while stirring diminishes the thickness of the aqueous boundary layer. Concerning membrane retention, this parameter is in general low (0-0.30). It has been observed that the membrane retention depends on the incubation time and also is related to the lipophilicity of compounds when they are in their neutral form. On the other hand, it has been demonstrated that the skin-PAMPA membrane is affected at basic pH values, so it is advisable to perform experiments below pH 8 to avoid damaging the membrane. The results shown here indicate good agreement between human skin permeability and skin-PAMPA permeability established under appropriate assay conditions.

## Figures and Tables

**Table 1.** log *P*_e_ values of the neutral form of the drugs, obtained from skin-PAMPA assays with and without stirring at different incubation times.

				log *P*_e_					
				With stirring			Without stirring		
Solutes	log *K*_p_ [18]	log *P*_o/w_ [19]	p*K*_a_	30 min	4 h	24 h	30 min	4 h	24 h
5-Fluorouracil	-6.82	-0.89	7.86 ^a^	-	-5.77(±0.02)	-6.16(±0.03)	-	-6.47(±0.22)	-6.07(±0.06)
Aminopyrine	-6.55	0.80	5.00 ^b^	-5.88(±0.07)	-5.65(±0.01)	-5.17(±0.06)	-5.79(±0.06)	-5.46(±0.06)	-5.22(±0.02)
Digitoxin	-8.15	2.83		-	-	-6.42(±0.27)	-	-	-6.34(±0.13)
Flurbiprofen	-4.72	4.16	4.19 ^c^	-2.98(±0.13)	-3.69(±0.02)	-	-3.54(±0.06)	-3.85(±0.03)	-
Griseofulvin	-6.44	2.18		-5.61(±0.01)	-5.25(±0.10)	-5.12(±0.02)	-5.65(±0.05)	-5.33(±0.07)	-5.20(±0.05)
Hydrocortisone	-7.22	1.61		-	-6.13(±0.03)	-6.04(±0.07)	-	-6.30(±0.18)	-6.14(±0.08)
Ibuprofen	-4.58	3.50	4.43 ^d^	-2.71(±0.04)	-3.61(±0.05)	-	-3.59(±0.08)	-3.65(±0.04)	-
Naproxen	-4.97	3.34	4.28 ^e^	-3.96(±0.12)	-4.19(±0.02)	-	-3.99(±0.08)	-4.24(±0.06)	-
Progesterone	-4.90	3.87		-3.25(±0.25)	-4.11(±0.10)	-4.59(±0.15)	-4.81(±0.06)	-4.88(±0.22)	-4.69(±0.12)

^a^ From reference [20]

^b^ From reference [19]

^c^ From reference [21]

^d^ From reference [22]

^e^ From reference [23]

**Table 2.** Membrane retention values of the neutral form of the drugs, obtained from skin-PAMPA assays with and without stirring at different incubation times.

				*R*_M_					
				With stirring			Without stirring		
Solutes	log *K*_p_ [18]	log *P*_o/w_ [19]	p*K*_a_	30 min	4 h	24 h	30 min	4 h	24 h
5-Fluorouracil	-6.82	-0.89	7.86 ^a^	0.00	0.04(±0.04)	0.08(±0.03)	0.00	0.01(±0.03)	0.05(±0.02)
Aminopyrine	-6.55	0.80	5.00 ^b^	0.00	0.00	0.05(±0.02)	0.00	0.04(±0.01)	0.05(±0.02)
Digitoxin	-8.15	2.83		0.00	0.22(±0.04)	0.29(±0.05)	0.00	0.09(±0.03)	0.25(±0.02)
Flurbiprofen	-4.72	4.16	4.19 ^c^	0.18(±0.03)	0.00	0.00	0.13(±0.02)	0.00	0.00
Griseofulvin	-6.44	2.18		0.06(±0.01)	0.19(±0.01)	0.24(±0.02)	0.03(±0.02)	0.16(±0.01)	0.20(±0.02)
Hydrocortisone	-7.22	1.61		0.02(±0.00)	0.01(±0.01)	0.08(±0.03)	0.00	0.04(±0.01)	0.07(±0.02)
Ibuprofen	-4.58	3.50	4.43 ^d^	0.22(±0.03)	0.00	0.00	0.19(±0.07)	0.09(±0.03)	0.00
Naproxen	-4.97	3.34	4.28 ^e^	0.13(±0.03)	0.00	0.00	0.09(±0.02)	0.04(±0.02)	0.00
Progesterone	-4.90	3.87		0.50(±0.04)	0.74(±0.01)	0.88(±0.01)	0.33(±0.01)	0.62(±0.04)	0.83(±0.02)

^a^ From reference [20]

^b^ From reference [19]

^c^ From reference [21]

^d^ From reference [22]

^e^ From reference [23]

**Table 3.** log K_p_ and log P_e_ experimental values of the neutral form of the drugs. Both *K*_p_ and *P*_e_ in cm/s units.

Solutes	log *K*_p_ [18]	log *P*_e_
2,4-Dichlorophenol	-4.30	-3.92± 0.00
2-Isopropyl-5-Methylphenol (Thymol)	-4.35	-4.01± 0.00
2-Nitro-p-phenylenediamine	-6.66	-5.25±0.02
3-Methylphenol (m-Cresol)	-4.89	-4.33±0.01
4-Amino-2-nitrophenol	-5.91	-4.59±0.02
4-Chlorophenol	-4.52	-4.270.02±
4-Ethylphenol	-4.53	-4.19±0.02
4-Hydroxyphenylacetamide	-6.89	-6.07±0.08
4-Hydroxy-methylphenylacetate	-5.26	-5.07±0.09
4-Nitrophenol	-5.33	-4.91±0.02
5-Fluorouracil	-6.82	-5.77±0.02
8-Methoxypsoralen	-5.12	-4.30±0.03
5,5-Diethylbarbituric acid (Barbital)	-7.29	-5.75±0.05
5-Ethyl-5-phenylbarbituric acid (Phenobarbital)	-6.68	-6.05±0.01
Benzoic acid	-5.68	-4.82±0.07
4-Hydroxybenzyl alcohol	-6.26	-5.85±0.01
Aminopyrine	-6.55	-5.67±0.03
Aniline	-4.94	-4.55±0.00
Benzyl nicotinate	-4.87	-4.16±0.02
Caffeine	-6.85	-5.45±0.02
Catechol	-5.87	-5.39±0.00
Cortexolone	-7.20	-5.45±0.03
Corticosterone	-6.84	-5.59±0.01
Cortisone	-7.38	-6.09±0.02
Dexamethasone	-7.27	-6.25±0.03
Diclofenac	-5.30	-3.79±0.02
Digitoxin	-8.15	-6.38±0.15
Estradiol	-5.61	-4.15±0.07
Fluocinonide	-6.33	-5.38±0.06
Flurbiprofen	-4.72	-3.69±0.01
Griseofulvin	-6.44	-5.28±0.06
Hydrocortisone	-7.22	-6.17±0.05
Hydroquinone	-6.31	-5.87±0.06
Hydroxyprogesterone	-6.30	-4.70±0.05
Ibuprofen	-4.58	-3.61±0.06
Indomethacine	-5.39	-4.40±0.04
Isoquinoline	-5.11	-4.20±0.01
Ketoprofen	-5.22	-4.68±0.03
Methyl 4-hydroxybenzoate	-5.12	-4.88±0.04
Methyl phenyl ether (Anisole)	-4.68	-4.34±0.04
Naproxen	-4.97	-4.19±0.04
o-Phenylenediamine	-6.70	-5.42±0.02
Piroxicam	-6.02	-4.67±0.05
Prednisolone	-7.91	-6.42±0.02
Progesterone	-4.90	-4.13±0.02
Testosterone	-5.54	-4.52±0.03

## Appendix

Equation (6) can be derived as follows for a drug equilibrated between an aqueous compartment and a membrane:

Therefore:

Because:

(6)

It should be noted that the derivation assumes that drug absorption to the filter material can be ignored. If this is not accurate then a constant can be added in order to improve the fit for less lipophilic compounds (equation 6b)

(6b)
